# Circulating leukocyte telomere length is highly heritable among families of Arab descent

**DOI:** 10.1186/1471-2350-13-38

**Published:** 2012-05-18

**Authors:** Omar S Al-Attas, Nasser M Al-Daghri, Majed S Alokail, Khalid M Alkharfy, Assim A Alfadda, Philip McTernan, Greg C Gibson, Shaun B Sabico, George P Chrousos

**Affiliations:** 1 Biomarkers Research Program, Biochemistry Department, College of Science, King Saud University, PO Box 2455, Riyadh, 11451, Kingdom of Saudi Arabia; 2 Center of Excellence in Biotechnology Research, King Saud University, Riyadh, Kingdom of Saudi Arabia; 3 Prince Mutaib Chair for Biomarkers of Osteoporosis, Biochemistry Department, King Saud University, PO Box 2455, Riyadh, 11451, Kingdom of Saudi Arabia; 4 Clinical Pharmacy Department, College of Pharmacy, King Saud University, Riyadh, Kingdom of Saudi Arabia; 5 Obesity Research Center, College of Medicine, King Saud University, Riyadh, Kingdom of Saudi Arabia; 6 Division of Metabolic & Vascular Health, Warwick Medical School, University of Warwick, CSRL, UHCW, Coventry, CV2 2DX, UK; 7 Center for Integrative Genomics, School of Biology, Georgia Institute of Technology, Atlanta, GA, USA; 8 Division of Endocrinology, Metabolism & Diabetes, University of Athens Medical School, Children's Hospital Aghia Sophia, Athens, Greece

**Keywords:** Telomere length, Heritability, Arabs, Ageing

## Abstract

**Background:**

Telomere length, an indicator of ageing and longevity, has been correlated with several biomarkers of cardiometabolic disease in both Arab children and adults. It is not known, however, whether or not telomere length is a highly conserved inheritable trait in this homogeneous cohort, where age-related diseases are highly prevalent. As such, the aim of this study was to address the inheritability of telomere length in Saudi families and the impact of cardiometabolic disease biomarkers on telomere length.

**Methods:**

A total of 119 randomly selected Saudi families (123 adults and 131 children) were included in this cross-sectional study. Anthropometrics were obtained and fasting blood samples were taken for routine analyses of fasting glucose and lipid profile. Leukocyte telomere length was determined using quantitative real time PCR.

**Results:**

Telomere length was highly heritable as assessed by a parent-offspring regression [h2 = 0.64 (*p* = 0.0006)]. Telomere length was modestly associated with BMI (R^2^ 0.07; *p*-value 0.0087), total cholesterol (R^2^ 0.08; *p*-value 0.0033), and LDL-cholesterol (R^2^ 0.15; *p*-value 3 x 10^-5^) after adjustments for gender, age and age within generation.

**Conclusion:**

The high heritability of telomere length in Arab families, and the associations of telomere length with various cardiometabolic parameters suggest heritable genetic fetal and/or epigenetic influences on the early predisposition of Arab children to age-related diseases and accelerated ageing.

## Background

The prevalence of chronic non-communicable diseases in the kingdom of Saudi Arabia, particularly in the greater metropolitan region of its capital Riyadh, has dramatically increased over the last decade [1]. Several conventional risk factors noted in adults, such as the continuing rise of a sedentary lifestyle [2] and excessive nutrition [3], have been established clearly in this area. Additionally, further studies suggest the presence of other risk factors, which appear common to both adults and children. These risk factors include changing sleeping habits [4], vitamin D deficiency [5,6] and dyslipidemia [7,8]. Such factors are known to significantly increase the pre-disposition to chronic non-communicable diseases among Arab adults. However the apparent observation that the same risk factors may now manifest earlier in Arab children indicates that modifiable environmental causes and genetic or epigenetic changes may also play pivotal roles in the increasing susceptibility to chronic non-communicative diseases, as well as a shortened life span and decreased quality of life.

Telomeres, tandem repeats of the DNA sequence TTAGGG extending over 6–15 kb at the ends of eukaryotic chromosomes, have been established as a modest biomarker of senescence and longevity [9,10]. Furthermore, strong evidence has linked decreased telomere length to several age-related diseases, suggesting that these chromosomal fragments may be a major functional node combining genetic, epigenetic and lifestyle factors of aging [11]. Heritability-wise, telomere length was noted as transmissible from parent to offspring in studies involving Caucasians with ischemic heart disease [12] and Caucasian families of Amish descent [13]. In the Arab setting, our earlier telomere length studies demonstrated cross-sectional inverse associations between telomere length and metabolic biomarkers of obesity and insulin resistance in both Arab adults and children [14,15]. These previous studies shed light on the possible role of telomere length as a marker for predisposition to age-related diseases in the Arab population. We recently demonstrated high heritability of adipocytokines and other cardiometabolic disease biomarkers in this family cohort [16]. However, whether or not telomere length remains a highly conserved heritable trait in a homogeneous Arab cohort has yet to be demonstrated.

## Methods

### Subjects

In this cross-sectional study, 254 subjects or 119 randomly selected, paired families (123 adults and 131 children) were selected using the Microsoft Excel function from the database of the Biomarkers Research Program in the RIYADH COHORT study. Out of the 119 families, only 2 children (both daughters) had a complete set of parents (father and mother). The rest were paired [a) father and son N = 40; b) father and daughter N = 35; c) mother and son N = 15; d) mother and daughter N = 25; and trio e) mother and 2 daughters = 1]. This study is a capital-wide, joint-collaborative study between the Ministry of Health and the Biomarkers Research Program (BRP) of King Saud University utilized for the screening of novel biomarkers in chronic non-communicable diseases from a roster of ~17,000 Saudi subjects (aged 1–80 years) recruited from randomly selected primary healthcare centers (PHCCs) in Riyadh, KSA.

Parents with and without existing illnesses and co-morbidities were included to avoid selection bias. Subjects were asked to complete general questionnaires, which included demographics and medical history. Formal inclusion into the study required written and informed consent obtained from parents, and assent from the children. Ethical approval was obtained from the Ethics Committee of the College of Medicine Research Center (CMRC) of King Saud University, Riyadh, KSA. This study sample was previously reported with the primary focus of determining the heritability of circulating adipocytokines [16].

### Anthropometrics, sample collection and analysis

Participating families were asked to return to their designated PHCCs following an overnight fast to obtain anthropometric data and blood biochemical analyses. The following measurements were taken: height (to the nearest 0.5 cm), weight (to the nearest 0.1 kg), waist and hip circumferences (measured using a standardized measuring tape in cm), as well as systolic and diastolic blood pressure. BMI was calculated as kg/m^2^. Adults having a BMI of ≥ 30 kg/m^2^ were considered obese, while overweight was defined as a BMI of > 25 but <30 kg/m^2^. For children, BMI was categorized based in the international age- and gender-specific criteria proposed by Cole and colleagues [17]. Fasting blood samples were extracted. Serum samples were obtained through centrifugation and transported to BRP for the routine analysis of fasting glucose and lipid profile using a chemical analyzer (Konelab, Finland).

### Quantification of telomere length

TL was obtained from leukocyte DNA isolated from whole blood using quantitative real time PCR utilizing an IQ cycler as previously described (Bio-Rad Laboratories, Hercules, CA, USA) [18]. In brief, the assay involved comparing the abundance of telomere DNA to an internal reference gene of invariant copy number for each sample and by further comparison of normalized values between DNAs of different sources. Two reference DNA samples (MRC5 and KE27) were used to construct standard curves of amplifications using GAPDH (fixed copy number reference gene) and telomere primer pairs. 1.68-fold serial dilutions were made for each DNA covering a range of 7.4 to 0.93 ng/uL. 10 uL aliquots of the diluted reference DNAs were dispensed to each of four replicate wells for each dilution, giving final quantities in the range of 74 to 9.3 μL of DNA per well. 15 μL of PCR cocktail containing 12.5 μL Taqman mastermix and 5 picomoles of each primer was added to each well and plates were cycled forty times at 95°C for 15 seconds and 56°C for 60 seconds. Plots of log [10] template quantity versus cycle threshold (Ct) showed linear relations across the entire dilution series for both primer pairs tested against both reference DNA samples. The slopes of the graphs were used to calculate average efficiency of amplification values for both primer sets [14,15].

### Statistical analyses

Analyses were performed using SPSS version 11.5 (Chicago, IL). Frequencies were presented as percentage (%), continuous variables were shown as mean ± standard deviation and median (inter-quartile range) for variables with non-Gaussian distribution. All subsequent analyses were performed using SAS/JMP version 4 (Cary, North Carolina). Pearson correlation and regression analyses were performed on standardized residuals from linear models of systolic and diastolic blood pressure, the anthropomorphic measures, or log-transformed measures of glucose and lipid profile that included covariates representing cohort, gender, generation, and age within generation. Significance was set at a p-value < 0.05.

## Results

General characteristics of parents and children are presented in Table 1. Table 2 reveals unadjusted parent-offspring regression for selected parameters. Among the parameters, BMI, weight, waist and hip circumference, as well as LDL- and total cholesterol were significant and, thus, considered highly heritable, even after adjustment for gender, generation and age within generation.

**Table 1:** General characteristics of the families studied

**Parameter**	**Parents**	**Children**
N	123	131
M/F	79/44	61/70
Obesity (%)	47.9	29.7
DM T2 (%)	30.6	0
Hypertension (%)	14.0	0
Age (years)	45.9 ± 10.1	9.37 ± 2.6
BMI (kg/m^2^)	30.0 ± 5.6	20.0 ± 5.5
Waist circumference (cm)	95.4 ± 13.3	62.8 ± 5.7
Hip circumference (cm)	104.9 ± 6	72.2 ± 15.0
Systolic BP (mmHg)	121.2 ± 12.6	99.8 ± 15.0
Diastolic BP (mmHg)	79.6 ± 9.1	66.0 ± 8.0
Glucose (mmol/L)	7.1 ± 3.7	4.9 ± 1.0
Triglycerides (mmol/L)#	1.5 (1.03-2.5)	0.88 (0.7-1.2)
Total Cholesterol (mmol/L)	5.2 ± 1.1	4.4 ± 0.9
LDL-Cholesterol (mmol/L)	3.4 ± 0.9	2.8 ± 0.7
HDL-Cholesterol (mmol/L)	0.86 ± 0.23	1.1 ± 0.29
Telomere length (kb)	6.53 ± 1.95	7.23 ± 2.06

**Note:** Data presented as mean ± standard deviation **; #** denotes Non-Gaussian variable, presented as median.

**Table 2:** Parent-offspring regressions for raw trait measures

**Parameters**	**Slope**	**R-squared**	**N pairs**	**P value**
BMI	0.26	0.08	113	0.0030
Body Weight	0.23	0.06	116	0.0099
Height	0.13	0.01	114	0.44
Hip circumference	0.35	0.11	113	0.0003
Waist circumference	0.26	0.06	114	0.0076
Glucose	0.09	0.01	111	0.25
Systolic BP	0.07	0.01	92	0.43
Diastolic BP	−0.09	0.01	92	0.30
Triglycerides	0.05	0.03	111	0.08
Total-Cholesterol	0.22	0.08	110	0.0033
LDL-Cholesterol	0.26	0.15	109	3 × 10^-5^
HDL-Cholesterol	0.09	0.01	111	0.45
Telomere length	0.64	0.26	42	0.0006

**Note:** P-value significant at p < 0.05.

### Telomere length

Telomere length was highly heritable as assessed by parent-offspring regression. A simple point estimate based on the slope of the linear regression of 42 single children and on a single parent noted heritability as *h*^*2*^ = 0.64 (*p* = 0.0006) (Table 2). Adjustments for gender, age and age within generation revealed that telomere length was also observed to be modestly associated with three other measures related to metabolic activity: BMI (R^2^ 0.07; *p*-value 0.0087), total cholesterol (R^2^ 0.08; *p*-value 0.0033), and LDL-Cholesterol (R^2^ 0.15; *p*-value 3 x 10^-5^) (Table 3). Separate analyses of paternal and maternal effect revealed difference in heritability (*h*^2^ = 0.59; *p*-value 8 x 10^-5^; *h*^*2*^ = 0.16; *p*-value 0.033 for paternal and maternal transmission, respectively). Naturally, TL was significantly different between the adult parents and their children (6.53 ± 1.95 versus 7.23 ± 2.06 *p* = 0.034). Telomere length was observed to decay at a rate of approximately 20 bp per year (not shown).

**Table 3:** Parent-offspring regressions for adjusted trait measures

**Trait**	**Slope**	**R-squared**	**N pairs**	**P value**
BMI	0.27	0.07	104	0.0087
Weight	0.22	0.07	107	0.0062
Height	0.17	0.02	106	0.12
Hip circumference	0.36	0.14	105	0.0001
Waist circumference	0.30	0.10	106	0.0008
Systolic BP	0.09	0.01	89	0.35
Diastolic BP	−0.02	0.00	90	0.78
Glucose	0.05	0.01	104	0.34
Triglycerides	0.05	0.03	103	0.09
Total-Cholesterol	0.24	0.08	104	0.0033
HDL-Cholesterol	0.09	0.04	105	0.50
LDL-Cholesterol	0.27	0.15	103	5 × 10^-5^
Telomere length	0.66	0.28	41	0.0003

**Note:** P-value significant at p < 0.05.

## Discussion

This study that telomere length is highly heritable in Arab families, which is consistent with several previous estimates based on sib pairs in a European Caucasian group [19]. While the estimate is robust as to the influence of age, since it remains unchanged after adjustment for gender, generation and age within a generation, it is certainly an over-estimate of the genetic and epigenetic contribution to telomere length for two reasons: First, it fails to account for shared familial environmental influences (including activity levels and diet) that may affect telomere decay rates. Second, classically, the slope of a single parent-offspring regression is often considered to be twice the heritability, since only half of that parent's genes are transmitted to the child. Doubling the value would result in a nonsensical heritability estimate greater than 100%. The stronger paternal effect on the offspring's TL confirms several large-scale studies and reinforces the significant father-to-offspring heritage of longevity trait [13,20].

Anthropometric parameters, such as BMI, body weight and waist and hip circumferences, were also highly heritable and related to telomere length in the present study, which means the susceptibility to obesity is evident in Arab families, and strengthens our previous finding that these measures of obesity, if deemed pathologic, predispose Arab children to accelerated ageing, as determined by shortened telomere lengths [15]. Similarly, these findings are compatible with the high heritability of variations in the circulating levels of adipocytokines in the Arab population [16]. However, whether the mean telomere lengths observed in this study for both parents and children are within the normal range cannot be determined, as there is no consensus as to what length is normal, probably because of the wide range of inter-individual variations and confounders observed in most studies [21].

Lastly, the association of telomere length to lipid parameters (total cholesterol and triglycerides) confirms our previous report [14]. Elevated levels of cholesterol and triglycerides are atherogenic and confer metabolic stress that damages DNA and dampens telomerase activity leading to telomere length attrition [22]. The link between cholesterol and telomere length is possibly due to increased cellular stress, which amplifies cell aging by damaging the telomerese by oxidation and by bringing cells to their maximum replicative capacity ultimately translating to shortened telomere length. This could also tie in with age-related innate immune pathway activation in adipose tissue and its link with sub-clinical systemic chronic inflammation [15].

This study has several limitations. Its cross-sectional nature limits our findings to mostly associations that are, at best, suggestive. Several confounders, such as diet and physical activity, were also not included. Nevertheless, the findings are strengthened by the homogeneity of the cohort studied. Dissemination of these results may have beneficial clinical implications for the ethnicity concerned, as they suggest that lifestyle changes and prevention are of utmost importance for both individual and public health.

## Conclusions

In summary, telomere length, which is a biomarker for ageing and age-related diseases, is highly heritable among Arab Saudi families. Our present findings, and the associations of telomere length to several cardiovascular and insulin resistance risk factors, as shown in our previous studies, confirm that the susceptibility of Arab children to premature ageing and age-related diseases is strongly inherited. Public health awareness campaigns focusing on families at increased risk for chronic diseases are highly recommended.

## Competing interests

The authors declare that they have no competing interests.

## Authors’ contributions

OSA, NMA conceived and carried out the study. AAF, MSA and KMA contributed in the study design, subject recruitments and data collection. PMT carried out sample analysis. SBS and GG performed statistical analysis. SBS and GPC participated in the design of the study and drafted the final version of the manuscript. All authors approved and read the final manuscript.

## Pre-publication history

The pre-publication history for this paper can be accessed here:

http://www.biomedcentral.com/1471-2350/13/38/prepub
